# Polymer-Based Device Fabrication and Applications Using Direct Laser Writing Technology

**DOI:** 10.3390/polym11030553

**Published:** 2019-03-22

**Authors:** Zhen-Lin Wu, Ya-Nan Qi, Xiao-Jie Yin, Xin Yang, Chang-Ming Chen, Jing-Ying Yu, Jia-Chen Yu, Yu-Meng Lin, Fang Hui, Peng-Li Liu, Yu-Xin Liang, Yang Zhang, Ming-Shan Zhao

**Affiliations:** 1 School of Optoelectronic Engineering and Instrumentation Science, Dalian University of Technology, Dalian 116024, China; zhenlinwu@dlut.edu.cn (Z.-L.W.); qiyanan_crystal@mail.dlut.edu.cn (Y.-N.Q.); yjy@mail.dlut.edu.cn (J.-Y.Y.); yu_jiachen@mail.dlut.edu.cn (J.-C.Y.); xiaomengzi@mail.dlut.edu.cn (Y.-M.L.); huifang@mail.dlut.edu.cn (F.H.); liupengli@mail.dlut.edu.cn (P.-L.L.); liangyuxin0318@mail.dlut.edu.cn (Y.-X.L.); yangzhang@dlut.edu.cn (Y.Z.); 2 State Key Laboratory of Integrated Optoelectronics, Institute of Semiconductors, Chinese Academy of Science, Beijing 100083, China; yinxiaojie@semi.ac.cn; 3 Henan Shi-Jia Photons Technology Co., Ltd., Hebi 458030, China; 4 Department of Electrical and Electronics Engineering, School of Engineering, Cardiff University, Cardiff CF10 3AT, UK; yangx26@cardiff.ac.uk; 5 College of Electronic Science and Engineering, Jilin University State Key Laboratory of Integrated Optoelectronics, JLU Region, Changchun 130012, China; chencm@jlu.edu.cn

**Keywords:** polymer-based laser direct writing, optical device, electromagnetics application, biology application

## Abstract

Polymer materials exhibit unique properties in the fabrication of optical waveguide devices, electromagnetic devices, and bio-devices. Direct laser writing (DLW) technology is widely used for micro-structure fabrication due to its high processing precision, low cost, and no need for mask exposure. This paper reviews the latest research progresses of polymer-based micro/nano-devices fabricated using the DLW technique as well as their applications. In order to realize various device structures and functions, different manufacture parameters of DLW systems are adopted, which are also investigated in this work. The flexible use of the DLW process in various polymer-based microstructures, including optical, electronic, magnetic, and biomedical devices are reviewed together with their applications. In addition, polymer materials which are developed with unique properties for the use of DLW technology are also discussed.

## 1. Introduction

Devices (here including devices/structures/patterns) based on low-cost polymer materials have been widely used in various fields, such as optics, electromagnetics, and biomedicine [1,2,3,4]. For the applications of optical devices, polymer-based optical materials have been commonly used in the fabrication of optical waveguides, polymer fibers, and micro-optics devices [5,6,7,8,9,10] due to the high optical transmission properties, good plasticity, and excellent mechanical stability. In addition, the excellent compatibility of the polymer material allows it to be combined with other materials with electro-optical or magnetic properties for the fabrication of organic electro-optical or magnetic components [11,12,13,14]. Due to the high electro-optic (EO) coefficients, high thermal stability, good flexibility, and low dielectric constant, polymer materials are also widely used for wide bandwidth, high-efficiency organic electro-optical modulation devices [15,16] as well as flexible electronic circuits and microelectronic devices [2,17]. For biomedical applications, various polymer materials have shown great potential for the fabrication of biomedical structures and devices. With the help of good biocompatibility and easy processing properties, polymer-based materials can be made into various shapes which provide a platform for biological reactions and allow different testing applications [18,19,20,21].

Direct laser writing (DLW) is a rapid manufacturing technology which was introduced in the 1980s during the development of large-scale integrated circuits. The initial applications were to use a laser beam to scan over a layer of photoresist material to produce a precision lens array or to make a mask on a photoresist-coated vulcanized glass-state semiconductor (CGS) [22,23]. The scanning accuracy that was achievable at that time was on the order of submicrons with a minimum line width of 1 μm [24]. Until the 1990s, DLW technology developed rapidly, and the direct writing process was improved and expanded to a large range of processing materials and applications [25,26]. In nearly ten years, with the urgent needs of micro/nano device processing technology in photochemistry, optoelectronics, and biomedicine, femtosecond (fs) DLW technology was investigated as a promising processing technology for device fabrication due to its three-dimensional (3D) processing capability, high processing resolution (far below the diffraction limit, the feature size is as small as 100 nm) [27,28,29,30,31,32], and the ability to fabricate various structures with, and even without, any mask [33,34,35,36].

**Figure 1:**
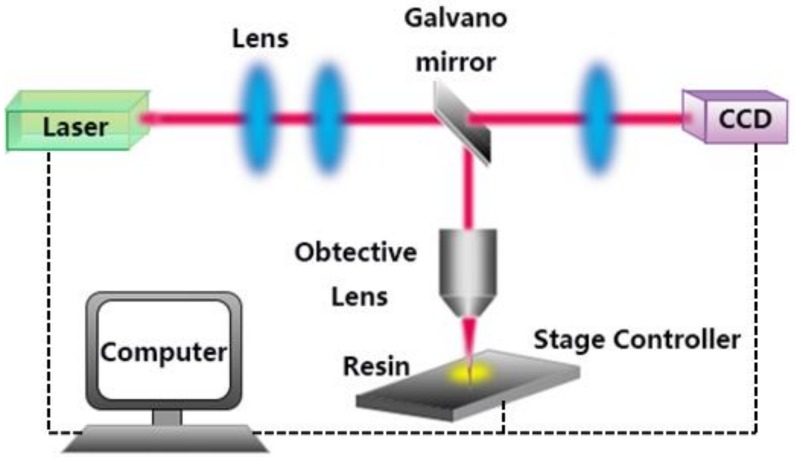
Schematic diagram of a DLW system.

The use of DLW technology for the polymer-based device fabrication, a combination of both advantages, has broadened the application of DLW method and have shown its unique superiority and practicality in the fields of micro-optics, microelectronics, as well as biomedicine. Figure 1 shows the schematic structure of the DLW system. Two photophysical/chemical mechanisms are adopted during the DLW process for the preparation of micro/nano structures, including photoablation and photopolymerization. The photoablation is an ion avalanche process in which the laser-focused area absorbs sufficiently high energy and detaches from the material in a molten or vaporized manner. Generally, micro-trench, porous microstructure, and patterning treatment of the polymer surface can be prepared by this method. Polydimethylsiloxane (PDMS), polyimide (PI), and poly(ethylene terephthalate) (PET), which are commonly used as substrates, can be applied in the ablation process [37,38,39,40]. The DLW process based on photopolymerization is also widely used in the fabrication of polymer-based micro/nano devices. Among them, DLW technology based on one-photon absorption (OPA) is suitable for manufacturing 2D patterns, which demands less laser power, while DLW technology based on two-photon absorption (TPA) is widely used to fabricate 2D, 2.5D, and 3D structures of arbitrary shape. Taking the two-photon absorption process as an example, the beam emitted from the femtosecond laser source is focused by a high numerical aperture (NA) objective lens (OL) into the interior of the material. In order to realize the two-photon polymerization, the intensity of the scanning laser is controlled to operate above the threshold intensity of the nonlinear absorption of the photopolymer materials. The position and the depth of the desired polymerization area are controlled by a stage controller, allowing the focused laser beam to scan over the polymer layer, and a high-resolution microstructure of certain shape can be obtained as the unexposed areas are properly removed after the development [39,41]. As materials used in this process need to have photosensitive properties, prepolymer resin containing a photoinitiator, such as IP-Dip or SU-8, are commonly used [27]. In addition, certain amount of dye molecules are added to the polymer substrate as a photosensitizer to enhance photopolymerization efficiency [42,43]. Besides the use of costly fs lasers for DLW, low-cost continuous wave (CW) lasers are also often used in the fabrication of micro/nano 2D/3D microstructures [44,45,46]. In addition, the researchers also proposed that a DLW system with continuous wave laser and a high NA objective to achieve low OPA of SU-8, which can achieve the resolution of several hundred nanometers [47,48].

In the fabrication of polymer-based devices using DLW, the commonly used laser emission wavelength is 400~800 nm, the excitation power ranges from a few milliwatts to several hundred milliwatts with the writing speed at least tens of micrometers per second. However, the resolution of the structure obtained is not only affected by complex chemistry (photo-reactions, dark reactions, diffusion, and chain growth), but also related to the selected parameters of DLW system, for instance, writing speed and laser power. In fact, in the manufacture of specific structures based on different applications, the determination of these two parameters needs to be investigated and accurately determined. During the experiment, both the polymerization threshold conditions of the selected polymer materials and the mechanical stability of the prepared structure need to be taken into account. In addition, not all polymers are suitable for DLW 3D structuring. As the material requirements for the 3D microstructure are that the prepared resin film needs to be thicker than the height of designed structure, only a few polymers can satisfy the formation of films with sufficient thickness and no structural defects [49]. For various device applications, component configuration, system parameters and the polymer material type need to be properly selected.

This paper reviews the latest research results of polymer-based devices fabricated using DLW technology. In this work, a review of polymer-based optical devices produced by DLW technique, including polymer fiber gratings, microresonators, microlenses, and optical waveguide couplers is given in Section 2. Section 3 introduces the fabrication of electromagnetic related devices using DLW method, such as electronic circuits, all-photon circuits, microcapacitors, and electro-optic modulator and magneto-photonic devices. Section 4 describes the application of DLW fabricated polymer-based microstructures in the field of bio-applications, including microfluidic channels, micro-networks, microneedles, and biomimetic actuation. Section 5 provides a brief introduction to the special polymer materials used in DLW polymer devices. Finally, the conclusions and outlook are given in Section 6.

## 2. Polymer-Based Optical Devices Fabricated by DLW

Polymer-based passive optical devices, such as polymer fiber Bragg gratings (POFBG), micro resonators, microlenses, and waveguide couplers, have been extensively studied due to their wide applications in the fields of optical communication, sensing, and imaging. This section describes the latest progress in the manufacture of polymer-based optical devices using DLW technology.

### 2.1. CYTOP POFBG by DLW

For the fabrication of polymer-based optical fiber gratings, polymethyl methacrylate (PMMA) was initially adopted. However, due to the high optical loss at 800 and 1500 nm regions, the use of PMMA for fiber gratings can be greatly limited. In addition, it was found that PMMA based optical fibers can be sensitive to ambient humidity, which will affect the variation of the fiber Bragg grating (FBG) modulation wavelength [50,51]. Later, thermoplastic olefin polymer of amorphous structure (TOPAS) gradually replaced PMMA due to its excellent humidity insensitivity, but its high transmission loss still became an obstacle for the application [52]. Cyclic transparent optical polymer (CYTOP) material, which is shown to have lower optical loss, became a good candidate for the fiber-related applications. In addition, the excellent transmission property of perfluorinated CYTOP polymer covers the visible to near-infrared region [53].

Recently, it has been proposed of using low loss CYTOP fiber with multimode gradient index for FBG inscription using plane-by-plane fs DLW process [41,51,54,55,56]. By controlling the change of the refractive index, grating length, and the width of the plane, the coupling of the FBG mode can be effectively optimized. This DLW method is convenient, flexible, and suitable for processing and production of multiple FBGs [41,55].

**Figure 2:**
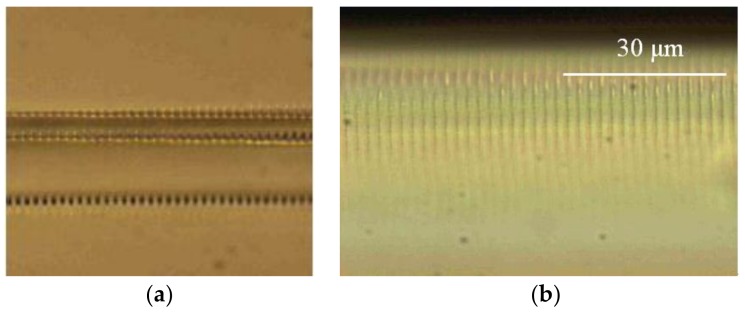
Microscopic image of FBG written by (**a**) PbP femtosecond laser; and (**b**) LbL femtosecond laser [54].

Lacraz et al. have investigated a plane-by-plane femtosecond DLW straight-through system for fabricating FBGs [54]. A commercially available CYTOP fiber is used with a core diameter of 62.5 μm and a cladding of 20 μm plus a polyester and polycarbonate outer jacket to protect it, and this outer layer is not removed during the direct writing process. The femtosecond laser system operates at 517 nm with a pulse duration of 220 fs and the repetition rate of the pulse selector is set as 1 kHz. The air bearing translation stage system provides precise biaxial movement of the fiber with respect to the laser beam which is focused using a long working distance microscope objective (50×). Each plane is written separately and can be either point-by-point (PbP) or line-by-line (LbL), allowing the grating size to be precisely controlled. The PbP method in Figure 2a can be easily written as a FBG, which can be clearly seen when viewed from the direction of laser energy deposition. In contrast, Figure 2b shows the engraved microscopic image of LbL FBG in CYTOP fiber multimode core with a line spacing of 2.34 μm.

**Figure 3:**
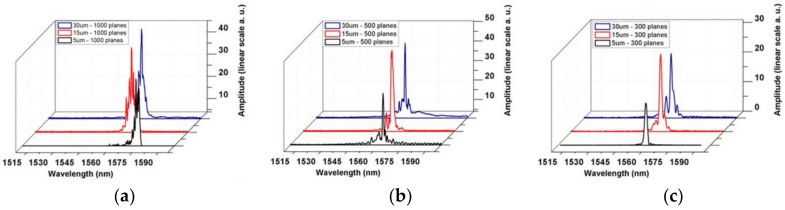
The grating reflection spectrum corresponding to 30, 15, and 5 μm wide with (**a**) 1000 planes; (**b**) 500 planes; and (**c**) 300 planes [51].

Theodosiou’s group has found that this DLW method can stimulate the strongest low-order mode by controlling the width and the number of inscribed planes to obtain a single-peak POF-FBG spectrum [51]. Figure 3 compares the reflection spectra of the various modes with different plane width and numbers of planes. It is considered that the best direct write parameters of FBG should be 300–500 planes (about 0.65–1.1 mm), and the plane width is 15 μm or less.

The discovery of the unimodal POF-FBG spectrum has led to more fabrication and application of this type of sensor. The researchers continued to write an FBG array directly in a 6 m long CYTOP fiber using optimized writing parameters. The 8 cm grating array consisted of six gratings that are physically discontinuous and spectrally separated in the 1500–1600 nm wavelength range. Each grating has a length of ~0.65 mm, a period of Λ = 2.2 μm, and a plane width of 15 μm. In the system for measuring the vibration response of metal beams, compared with the silica FBG sensor array, the sensitivity of the grating sensor fabricated using DLW method can be increased by 6 times. Using the same straight-through principle and parameter range, this kind of FBG can be more widely applied to different fields. Theodosiou et al. designed a 2 m long FBG array as a quasi-distributed sensor and applied to the health monitoring of the carbon cantilever beam of the helicopter tail rotor [55]. Small weights are attached to different positions on the cantilever beam, and the dynamic modal shape of the beam can be extracted to detect damage. The strain, temperature, and relative humidity responses of the POF-FBG are carefully calibrated to eliminate the cross-sensitivity. The same group continued to process the chirped grating and the FBG-Fabry Perot (FBG-FP) cavity using the low-loss CYTOP fiber [56]. The chirped grating consists of 2000 cycles, and the chirp is ~2.22 nm/mm. The F-P cavity is made of two identical 4th POFBG separated by a certain distance.

### 2.2. Polymer-Based Micro-Resonators by DLW

This section introduces the design and fabrication of micro-resonators using DLW method and their latest application progress as micro-resonant sensors and microlasers.

#### 2.2.1. Micro-Resonators for Sensors

Micro-resonant sensors, such as micro-rings [57], micro-disks [58], and microspheres [59], have a waveguide structure that can greatly change the wavelength of the transmission spectrum due to the numerous resonances of light passing through it. The DLW method are commonly used to prepare polymer-based micro-resonator sensors with a high-quality factor (*Q*-value) in various structures.

**Figure 4:**
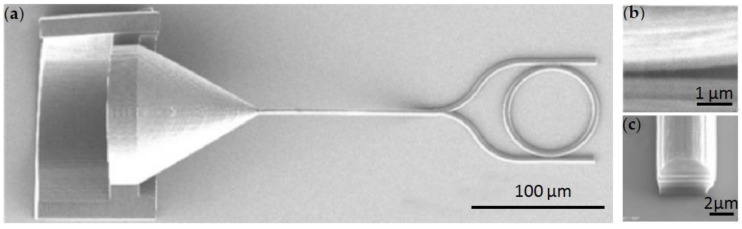
(**a**–**c**) SEM image of resonant micro-ring by DLW [60].

Wei et al. proposed a micro-ring resonator sensor fabricated by DLW technology (Nanoscribe GmbH) [60]. The fused silica substrate is bonded to the sample stage with the bottom surface coated with photoresist coverage (IP-Dip, refractive index is 1.54). The entire DLW process is performed in immersion mode, where the microscope objective is immersed in IP-Dip to achieve high resolution. Figure 4 shows the fabricated micro-ring resonator sensor.

The micro-ring resonator is integrated with an erbium doped fiber micro-ring laser (FRL) which can be used as an adaptive high frequency ultrasonic detector. A substrate having a resonant sensor integrated with the SMF is mounted at the bottom of the water tank and immersed in water. An ultrasonic wave which directs to the micro-ring can be generated using a piezoelectric ultrasonic transducer with a center frequency of 10 MHz. Acoustic waves that cause strain or deformation of the micro-ring resonator will shift the resonant wavelength, while the shift in the micro-ring resonator reflection spectrum can be used to reflect the frequency of the ultrasonic signal.

**Figure 5:**
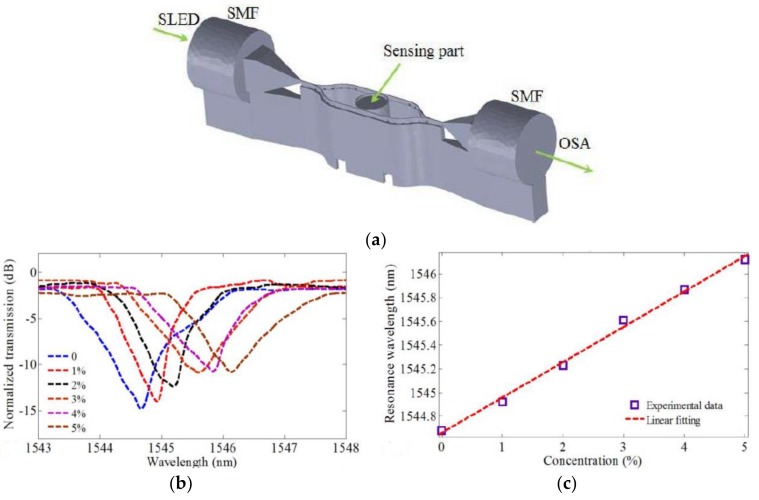
(**a**) Micro-cylinder resonance sensor; (**b**) transmission spectrum of the sensor for different concentrations of NaCl solution; and (**c**) relationship between wavelength in (**b**) and concentration of NaCl solution [61].

Similar DLW process used for resonant sensor structure preparation is proposed by Wei et al. to measure the refractive index of liquid [61]. The sensor structure consists of two tapered waveguides with a length of 110 μm and two Y-shaped waveguides. The tapered waveguide are coupled with single mode fiber (SMF), while the Mach–Zehnder interferometer (MZI) is coupled with the micro-cylinder for sensing purposes. The micro-resonator sensor is coupled to the two straight waveguides of the MZI, as shown in Figure 5a. The gap between the waveguide and the outside of the cylinder is designed to be about 0.2 μm for the strongest coupling. The micro-resonator sensor achieves a Q value of up to 6400 and a resonance loss of 15 dB. The strong interaction between the medium and the whispering gallery modes (WGMs) causes the shift in resonant wavelength, which corresponds to the refractive index change in the surrounding medium of the micro-cylinder. The high sensitivity response is approximately 154.84 nm/RIU, as shown in Figure 5b,c.

**Figure 6:**
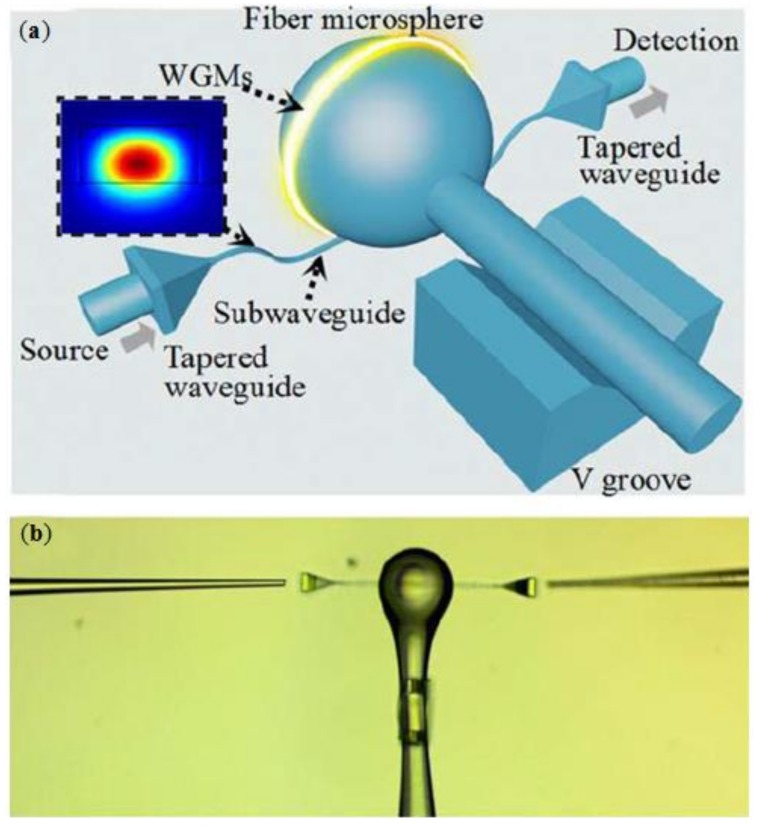
(**a**) Schematic diagram of coupling of 3D waveguide by DLW and resonant microsphere; and (**b**) the SEM image of the coupled sensor [6].

Normally, the WGMs is transmitted in a microcavity by evanescent coupling with a tapered single mode fiber [61], in order to enhance the stability of the effective coupling between the WGM resonant sensor and the inlet-outlet waveguide, a new structure is investigated by Wei et al. [6]. It uses the polymer waveguide fabricated by the DLW method for evanescent coupling with silica WGM microspheres, which is able to maintain the high Q factor. A 3D polymer substrate is fabricated using a 780 nm femtosecond laser. Two tapered waveguides and a V-shaped groove structure with a cross section of 25 μm × 25 μm are prepared, and the V-shaped groove connected to a straight sub-waveguide is used to guide light to couple into the resonator and support the silica microspheres, as shown in Figure 6. Tapered waveguide is used to couple with WGM to provide better long-term mechanical stability for sensing and laser applications.

#### 2.2.2. Micro-Resonators for Laser

**Figure 7:**
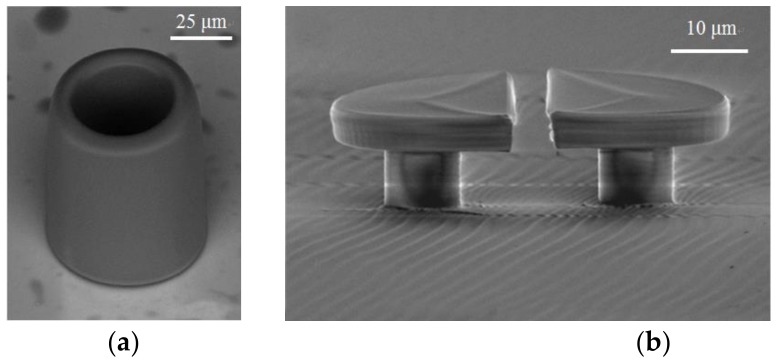
The SEM image of (**a**) WGM microcavity laser; and (**b**) tunable WGM laser [42,62].

Another application of micro-resonators fabricated using DLW process is in the field of micro-lasers. As shown in Figure 7a, Tomazio et al. reported a WGM micro-cavity laser doped with Rhodamine B dye in an acrylic-based polymer hollow micro-column [42]. The micro-cavity is manufactured by a femtosecond laser using a Ti:sapphire oscillator. With the help of the finely-controlled laser beam, the hollow microcylinder can be formed with an outer diameter of 50 μm, a side wall thickness of 6.5 μm, and a height of about 80 μm. It is excited by free-space pulse at 532 nm to achieve threshold pump energy as low as 12 nJ. Seigle et al. proposed another novel tunable WGM laser, which is realized by DLW. A disk is constructed on an elastomer PDMS substrate, which is composed of two opposite half disks with an intermediate air gap, as shown in Figure 7b [62]. By mechanically stretching the elastomer laterally to elongate or compress the substrate, the width of the gap, i.e., the resonant wavelength, can be controlled. The experiments have shown that the spectral tunability of the laser mode is more than three times higher than the free spectral range.

**Figure 8:**
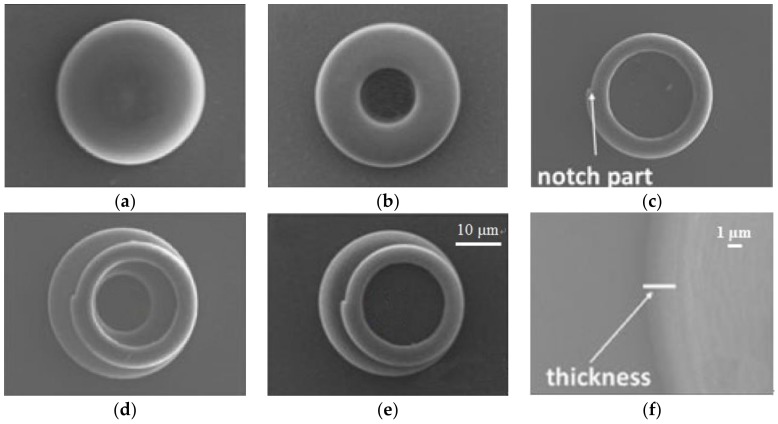
The SEM image of three different construction microlasers with (**a**) circular disk; (**b**) circular ring; (**c**) spiral ring; (**d**) a spiral ring stacked on a circular ring; (**e**) a spiral ring stacked on a circular disk; and (**f**) the tilt-view magnified SEM image of the circular ring in (**d**) [43].

In order to obtain a single-mode unidirectional laser, it has been reported to use the dye RhB-doped negative photoresist SU-8 to write a micro-laser on a 3D sheet with fs DLW [43]. It consists of two micro-rings and spirally stacked on top of each other, see Figure 8d,e, to achieve a unidirectional and single-mode output with a laser threshold of 60 μJ/cm^2^ at room temperature. The microcavity is pumped by a picosecond frequency doubling Nd:YLF laser with a wavelength of 532 nm. Several different polymer microcavities are fabricated directly on the narrow band filter substrate, as shown in Figure 8. The result shows that the ring microcavity can provide a low threshold laser emission, while the spiral ring microcavity can play the roles of mode filter and output port. In addition, DLW technology can be used to process photoresist containing nano-diamond (including the nitrogen vacancy center). The fabricated micro-resonator can then be fully applied to the quantum photonic circuit as a 3D quantum emitter [63].

### 2.3. Polymer-Based Microlenses by DLW

**Figure 9:**
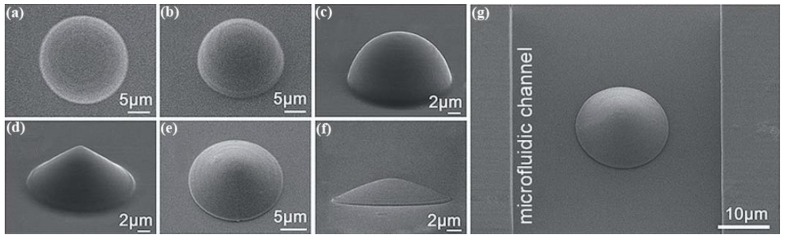
The SEM images from different view angles of (**a–c**) Spherical microlens and (**d–f**) aspheric hyperboloid microlens; and (**g**) SEM image of the microfluidic channel integrated with microlens [65].

Microlenses, especially microlens arrays, have been widely studied and applied due to their unique focusing and imaging characteristics, as well as their high integrity property with tiny volume and light weight. Polymer materials, such as SU-8 and PDMS, are adopted to fabricate microlens arrays using the DLW method. In [64], a 405 nm all-fiber laser is selected and spliced to a single mode fiber with small mode field diameter to form microlenses on the prepared SU-8 polymer film. The energy density of the laser writing was finely controlled between 90 and 600 J/cm^2^, to form the certain diameter of the lenticular structure, which was linearly increased with laser energy density. The method is suitable for microlens fabrication with constant focal length. Lu et al. used femtosecond DLW technique to produce PDMS microlenses that exhibit controllable and reversible adjustment of the focal length as the solution environment changes [65]. It depends on the sensitivity of PDMS to organic solvents. The PDMS microlens can be programmed according to the preset parameters to produce spherical microlenses and aspheric hyperbolic microlenses. In this work, thioxan-9-ketone, as a photosensitizer, is added to PDMS (1:1000 (*w*/*w*)) to prepare the photopolymer material. The laser beam is focused on the PDMS photopolymer using an oil immersion objective with a high numerical aperture (60×, NA = 1.4). The PDMS microlens is fabricated by scanning the laser focus point with an exposure time of 1000 ms. The SEM images in Figure 9 show the spherical microlenses and aspheric hyperbolic microlenses prepared by the DLW process.

**Figure 10:**
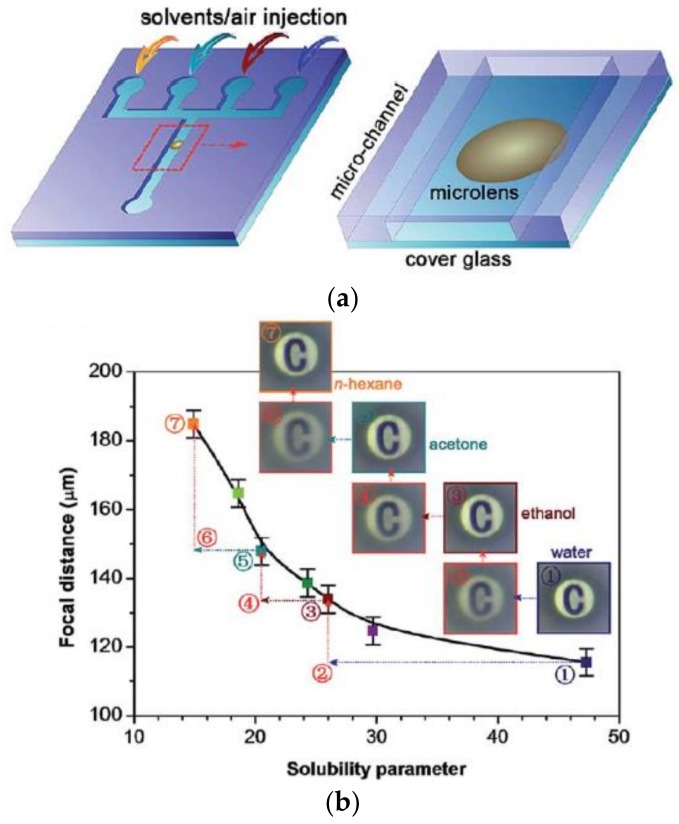
(**a**) Schematic diagram of microfluidic channel integrated with hyperboloid microlens; and (**b**) the relationship between the focal length of the microlens and the different solutions in the channel [65].

Since the PDMS material is very sensitive to organic solvents, Figure 10a shows a schematic diagram of a PDMS hyperboloid microlens with a radius of 20 μm and a height of 3.5 μm integrated into the microfluidic channel. The focal length of the PDMS hyperbolic microlens can be tuned by injecting different solvents in the channel, thereby changing its imaging characteristics. Figure 10b shows a significant change in the focal length of the microlens under the stimulation of organic solvents with different solubility parameters. The focal length of the obtained PDMS microlens can be dynamically altered between 112 and 185 μm.

However, with the continuous development of adjustable microlens, more researchers have proposed new ideas. He et al. investigated a polymer-based parabolic microlens array with homogeneous LC alignment layers written by a femtosecond DLW technology. The liquid crystal (LC) mixture, E7 (Merck), is filled into the fabricated microlens array. Due to the electronic property of the LC molecules, the focal length of the microlens array can be adjusted by the applied voltage [66].

**Figure 11:**
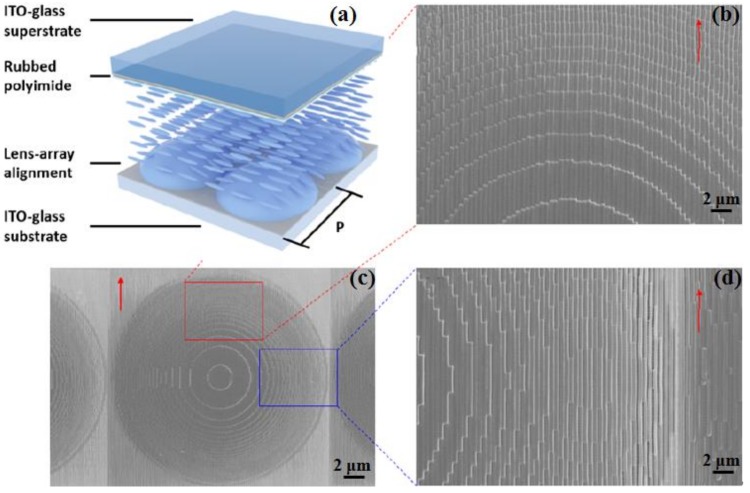
(**a**) Schematic diagram of the adjustable microlens array; and (**b–d**) the SEM images of the prepared microlens array [66].

A 16 × 16 polymeric parabolic shaped microlens array with a single microlens size of 120 × 120 μm^2^ is fabricated on an indium tin oxide (ITO) coated planar substrate using the DLW technique [66]. Figure 11 shows a schematic diagram and SEM images of an adjustable microlens array prepared in this work. After the photoresist IP-Dip is dropped on the single-sided ITO substrate, the objective lens (63×, NA = 1.4) is directly immersed in the IP-Dip. In the writing process, individual microlenses are written in divided sub-regions. In one sub-region, the microlens is formed layer-by-layer as the focal length of the laser is scanned. Each layer consists of 700 nm periodic grooves to provide anchoring force to LC molecules [67]. An ITO-glass superstrate with polyimide layer parallel to the trench is used, while the gap between ITO layers are sufficiently filled with LC mixture E7 (Merck). In order to control the spatial orientation of the LC by voltage modulation, the phase profile of the parabola-shaped microlens, i.e., the focal length, can be changed accordingly. In addition, DLW technology can also be used to produce composite lens systems with multiple refractive surfaces that exhibit high optical imaging quality with resolutions up to 500 lp/mm [68].

### 2.4. Polymer-Based Optical Waveguide Couplers by DLW

**Figure 12:**
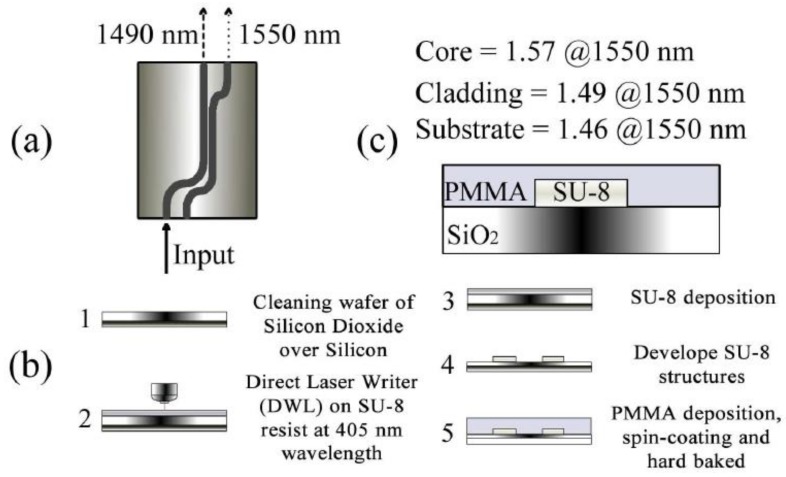
(**a–c**) Schematic diagram and manufacturing steps of directional coupler [71].

This section describes two different configurations of optical waveguide couplers, including duplexers based on directional coupling structures and Y-beam optical waveguide splitters. DLW technology is also suitable for the fabrication of optical waveguide couplers. The SU-8 polymer material has a high refractive index and a transmittance of over 95% (λ > 400 nm), which makes it suitable for communication applications with the working wavelength between 1310 and 1500 nm [69]. Directional coupling duplexers are commonly used to separate the received video signals of the 1490 nm channel and the 1550 nm channel in a fiber-to-the-home (FTTH) system [70]. Ramirez et al. proposed a directional coupler prepared using DLW technology with 405 nm laser [71]. The H-nu 470 photoinitiator-doped SU-8 photoresist, used as the core layer, is processed by DLW on the SiO_2_/Si bottom cladding. PMMA is deposited as a top cladding on top of the optical waveguide structure. The steps and parameters of the manufactured optical duplexer are shown in Figure 12. The fabricated directional coupler consists of two single-mode rectangular waveguides with a width of 1.7 μm, a height of 600 nm, and a spacing of 1.5 μm at the coupling area.

**Figure 13:**
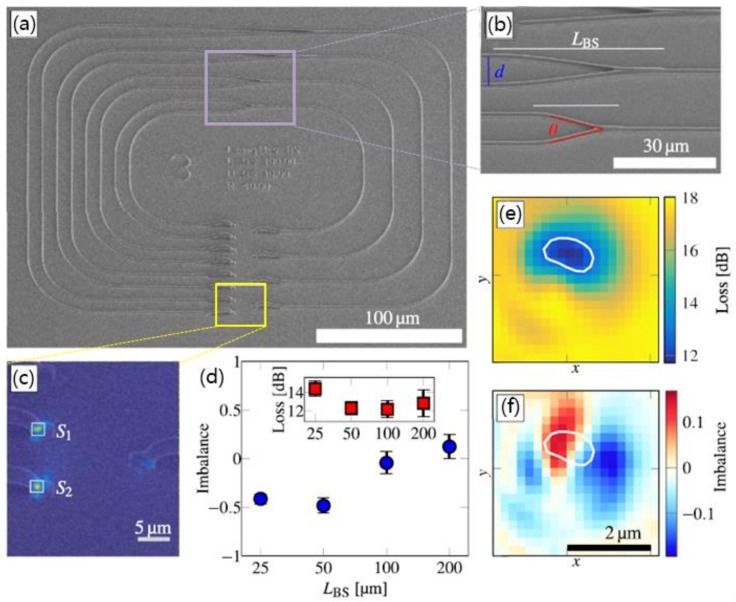
(**a**,**b**) The SEM image of the runway with several Y beam splitters with different separation lengths and the enlarged view of the Y area; (**c**) intensity distribution of the input and output at maximum transmission; (**d**) dependence between inbalance, insertion loss and separation length LBS; and the dimensional maps of (**e**) insertion loss and (**f**) imbalance of the longest waveguide shown as a function of the in-coupling position (x, y) [72].

In addition, another commonly used optical waveguide device, the Y-beam splitter coupler, can also be manufactured using DLW process. Landowski’s group studied a low-loss and high-integration density waveguide with an overall size of 300 μm × 300 μm written on the negative photoresist EpoClad 50 polymer material [72]. The writing system uses an ultrashort pulse laser with 780 nm center wavelength, 100 to 200 fs pulse length, 80 MHz repetition rate. Figure 13 shows the Y-shaped waveguide beam splitter fabricated in a stadium runway-type 3D structure. Optical properties, such as insertion loss and coupling imbalance of all five beam splitters are characterized. Due to various separation angles, the insertion loss decreases as the length of the beam splitter increases. It is proposed that these fabricated devices can be possibly applied in quantum optical network with high integration density [72]. Polymer waveguide fabricated by DLW can also realize photonic wire bonding, which is widely used in the interconnection of multi-chip integrated photonic circuits [73].

## 3. Polymer-Based Electro/Magnetic Devices by DLW

The unique properties of polymer materials not only suitable for the manufacture of optical devices, but also plays an important role in the fabrication of electromagnetic-related devices. With the help of the DLW method, polymer materials have been investigated and applied to prepare various electromagnetic structures, such as electronic circuits, photonic circuits, microcapacitor devices, electro-optic modulation devices, and magneto-optical devices with high processing resolution.

### 3.1. Electronic Circuits by DLW

**Figure 14:**
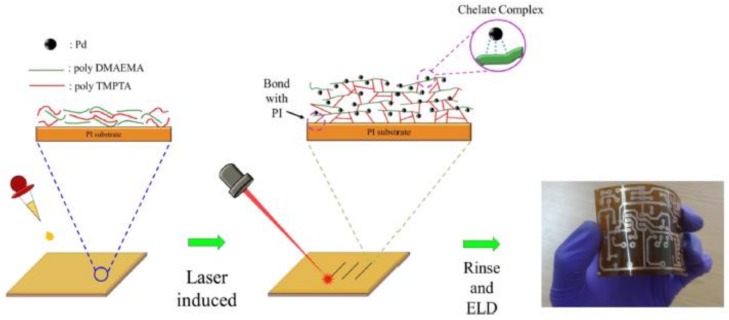
Schematic diagram of the fabrication of Cu-based electronic circuits on PI substrates by thermally curing Pd inks [45].

Recently, the DLW process has been introduced to achieve selective metallization on polymer substrates for fabricating flexible electronic circuits. For most of the flexible electronic circuit devices, metallic copper has been used as an electrode material for interconnection with polymer substrate because of its high electrical conductivity. Wang et al. proposed a simple selective metallization method using polyimide (PI) as the substrate material in [45] due to its high thermal stability and low dielectric constant [74]. A heat-curable Pd complex ink is proposed, which can promote adhesion between the Cu coating and the PI substrate. The electronic circuit fabrication process is shown in Figure 14. In this work, a 405 nm continuous wave laser is selected for the direct writing process. The laser beam is focused on the surface of the PI substrate using a beam expander and a microscope to form a spot with the size of 50 μm in diameter. The Pd complex ink spin-coated on the PI layer is cured and reduced selectively by the laser beam to form a polymer-grafted substrate. Then, the polymer-grafted PI is immersed in an electroless copper plating bath to deposit the copper electrode, which has the minimum line width of approximately 50 μm. This method provides a successful Pd catalyst patterning for electroless Cu deposition with high resolution.

The Pd complex material, which is used as the interconnected catalyst, is relatively high in cost for these applications. Ratautas et al. found that a low-cost composite material, multi-walled carbon nanotube (MWCNT)-doped polypropylene (PP), can be used as a laser-induced selective metallization material [75]. The low dose of MWCNT additive (2%, *w*/*w*) can reduce the cost of the material by at least 2–5 times. In the experiment, the mixture of PP and MWCNT is injection-molded at a certain mass ratio to form the substrate for selective metallization process. The optimized parameters of the nanosecond pulse laser, determined as 3 m/s of scanning speed and 100 kHz of pulse repetition rate, are used to achieve a Cu pattern with a minimum wire width of less than 22 μm.

Shin et al. investigated another DLW technique, i.e., the polymer-based laser ablation method, to prepare the designed pattern on the substrate surface [76]. In this study, a metal polylactic acid (PLA) filament containing micro-sized metal particles is used as the substrate material. The polymer can be removed more efficiently using an optimized irradiation laser pulse. The Cu particles contained in PLA can be directly connected to each other to form a metal pattern with high electrical conductivity of 0.15 Ω/cm and a total length of 50 mm. Certainly, the DLW technology for preparing metal-containing microstructures is not limited to the fabrication of Cu-based electronic circuits, but it can also be fully studied and applied to the fabrication of complex 3D metal nanoparticles including micro-nanostructures [77,78].

### 3.2. Photonics Integrated Circuits by DLW

DLW technology can also be used to manufacture all-organic photonics integrated circuits (PIC). In order to make electronically active organic PICs, it is important to integrate the waveguide structure with electro-optical materials. LC is a fluid substance with optical anisotropy. If an external electric field is applied, the orientation of the LC unit can be changed, while the applied field is removed, the LC molecular sequence will switch back to its original orientation [79]. Cano-Garcia’s group designed a PIC device which combines the polymer optical waveguide with the LC material and can be used as a switching polarizer in the visible region (630 nm) [11]. This work uses DLW method to write a directional trench at the certain position of the epoxy polymer waveguide and fill LC material inside.

**Figure 15:**
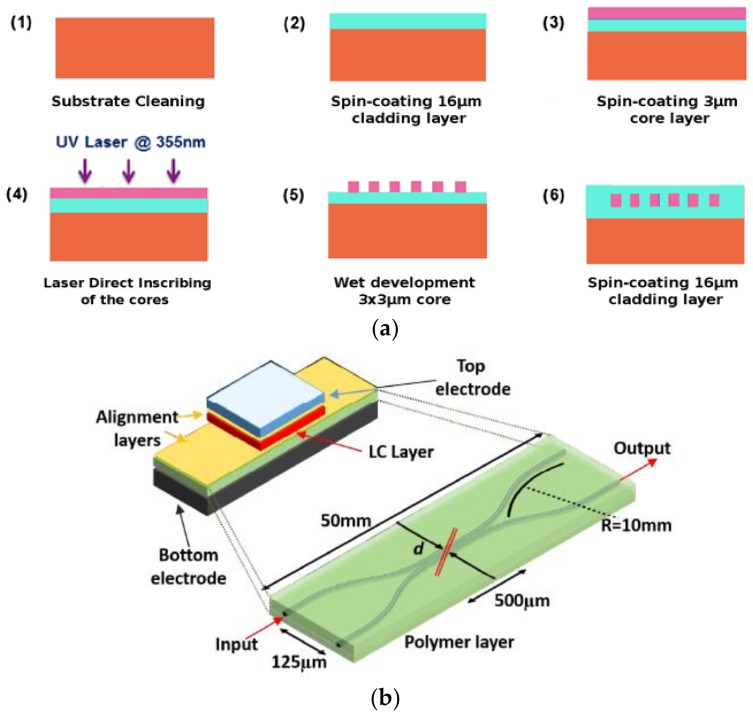
(**a**) The manufacturing process of waveguide structure; and (**b**) the structure and size parameters of PIC [11].

The manufacturing process of the waveguide is shown in Figure 15a. The polymer materials, epoxy resins (*n*_core_ = 1.590, *n*_clad_ = 1.578), are used for DLW process to obtain waveguides with a rectangular cross-sectional area of 3 μm × 3 μm (*n*_TE_ ≈ *n*_TM_ ≈ 1.585). Figure 15b shows the structure and size parameters of the PIC. A metallized silicon wafer is used as the bottom electrode and a transparent ITO-coated glass is adopted with a SiO_2_ alignment layer as the top electrode. A 20 μm groove is etched through the coupling region of the waveguide structure using the laser ablation method. The polymer layer is first deposited with a SiO_2_ alignment layer by the oblique evaporation, which can adjust the LC alignment orientation [80]. And then the LC material MDA-98-1602 is filled into the trench. It is found that, as *V*_off_ is applied, the TE polarization is directed to the output port, while *V*_on_ is applied, and the TM polarization is obtained.

### 3.3. Micro-Supercapacitors by DLW

**Figure 16:**
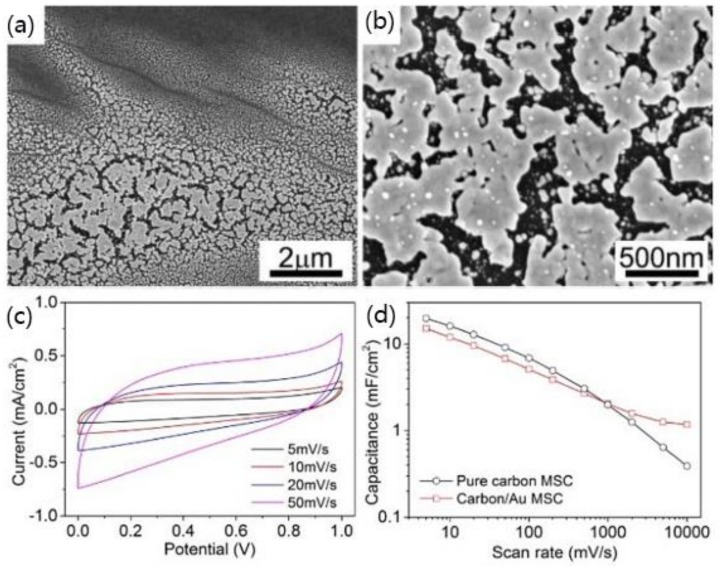
(**a**,**b**) The SEM images of carbon/Au composite electrode; and (**c**) CV curves of carbon/Au MSC; (**d**) Comparison of surface capacitance of pure carbon MSC and carbon/ Au MSC [83].

Micro-supercapacitors (MSCs) are small energy storage units that can realize wireless charging and discharging, which are designed to have large storage capacity, excellent recyclability, light weight, and are suitable for circuit integration [81]. The application of DLW technology for the manufacture of MSCs generally includes two directions [82], one is to use DLW to reduce GO to obtain rGO film with high conductivity; another method is to realize surface carbonization of the polymer film by DLW to form a high porosity carbon material. Cai et al. proposed a method of preparing a carbon-based MSCs with high surface capacitance using a low-cost 405 nm wavelength blue-violet semiconductor laser [83]. The DLW process is performed on a 125 μm polyimide film in argon environment to reduce the effect of oxidation. A carbon/Au composite electrode is prepared using a two-step DLW method to improve conductivity and charge-discharge performance. The first step is to carbonize the PI surface using DLW, while the second step is to coat a layer of Au nanoparticle ink and to let the laser irradiate the same position. Figure 16 shows the surface topography and performance of the carbon/Au composite structure. The surface capacitance can reach 1.17 mF/cm^2^ at a scan rate of 10,000 mV/s.

**Figure 17:**
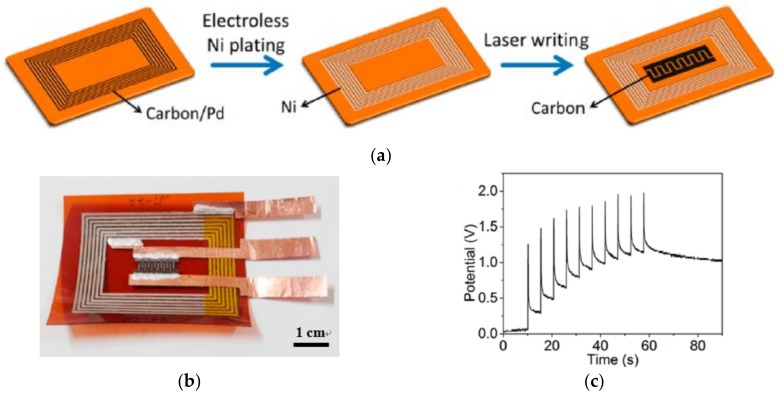
(**a**) Schematic representation of a device in which a carbon/Ni composite outer coil is integrated with an internal carbon MSC; (**b**) manufactured integrated wireless charging and storage device; and (**c**) the changes of the potential between two MSC electrodes as several wireless pulses are applied [84].

Using the same 405 nm blue-violet CW semiconductor laser device, in another work [84], Cai et al. prepared a carbon structure by DLW on PI substrate and then used electroless nickel to obtain a carbon/Ni composite electrode to achieve wireless charging and energy storage. First, a thin layer of polyvinylpyrrolidone (PVP)/PdCl_2_ is coated on the surface of the PI, after the DLW process, non-irradiated PVP/PdCl_2_ is removed to produce a carbon/Pd pattern. The carbon/Pd pattern is selectively metallized by electroless Ni plating to obtain a carbon/Ni composite structure. Figure 17a shows the process of preparing the integrated MSC device. The device can be divided into two parts. The first part is a carbon/Ni composite outer coil for collecting energy from electromagnetic waves, which is composed of seven spiral rectangular carbon/Ni wires with the width of 600 μm. The gap between two adjacent lines is 400 μm and the total coil size is 4 cm × 3 cm, see Figure 17b. The second part is an internal carbon-based MSC for energy storage at the center of the circuit, which is prepared by direct laser writing at 157 mW in argon protection environment. Figure 17c shows the good charging property and storage capability of the fabricated MSC devices because the potential between two MSC electrodes can increase fast to about 2 V within 60 s by applying several pulses.

### 3.4. Electro-Optic Waveguide Modulator by DLW

Organic electro-optic (EO) materials have been developed for use in electro-optical modulation devices due to their high EO coefficients and low optical absorption in the visible and infrared ranges [85]. Nitiss et al. investigated an EO waveguide modulator based on all-organic materials with a Mach–Zehnder interference structure using DLW technology [86]. SU-8 is selected as the waveguide core layer material of the modulator with low propagation loss, while DMABI-Ph6-doped PMMA is used as the cladding material due to its nonlinear electro-optic properties. Adjusting the concentration of DMABI-Ph6, a nonlinear optical chromophore material, can change optical property of the waveguide and avoid formation of crystallites which may affect EO efficiency. It is reported that the optimum doping concentration of DMABI-Ph6 was determined to be 30 wt % to achieve the best EO efficiency with single mode operation.

**Figure 18:**
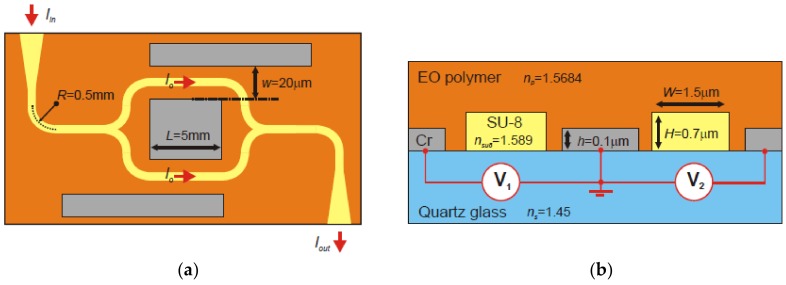
Dimensional parameters of the designed electro-optic modulator and its (**a**) top view; and (**b**) side view [86].

Figure 18 illustrates the structure of the prepared electro-optic modulator. A positive photoresist MEGAPOSIT SPR 700 polymer is used and spin-coated on the quartz substrate. A 365 nm laser is selected as the working laser to form electrode pattern on the substrate by mask exposure. Three 100-nm thick Cr electrodes are sputtered on the photoresist using an AUTO 306 thermal evaporator. After removing the unexposed positive resist, a 0.7 μm thick SU-8 layer is spin-coated as the core layer. The MZI waveguide structure is fabricated by DLW technology. The estimated EO coefficient is around 0.20 pm/V, which is very close to the typical value from the devices prepared using hybrid sol-gel EO polymer [87]. In order to obtain a higher EO coefficient, it is also necessary to synthesize novel EO polymers with stronger optical nonlinearity in the desired wavelength range, which can be an interesting field for further research.

### 3.5. Magnetophotonic Microdevices by DLW

Micromachines require mechanical movements of certain components. Many solutions have been proposed on how to fix or drive micro-mechanical units to realize designed functions, among which the magnetic driving has become a good candidate as a safe remote control method [88]. By using low-cost DLW technique, Nguyen’s group has prepared various magneto-photonic microdevices which can be driven by magnetic force [89]. The DLW method adopted in this work is based on the low one photon absorption (LOPA) effect using polymer-based nanocomposites with SU-8 2005 photoresist and Fe_3_O_4_ nanoparticles. The optimum doping concentration of Fe_3_O_4_ nanoparticles is determined as 2 wt % in order to reduce the agglomeration of nanoparticles and generate strong response to external magnetic fields.

**Figure 19:**
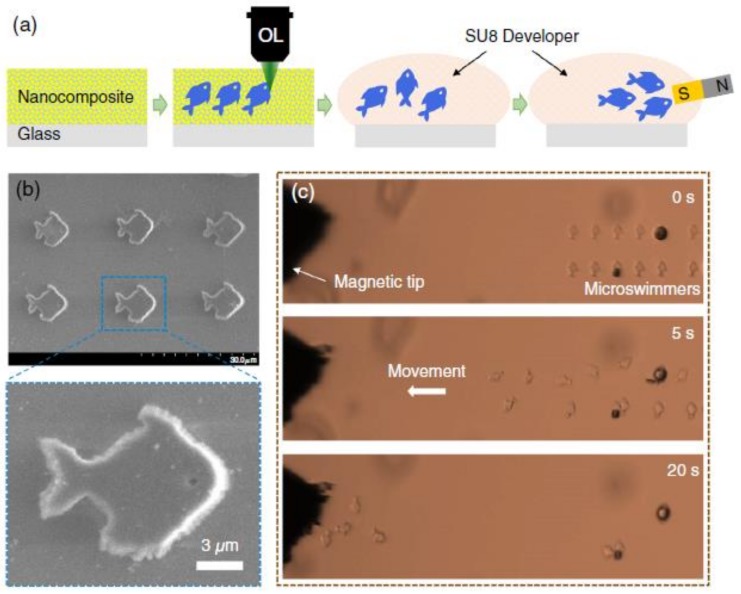
(**a**) Schematic of the fabrication of micro-swimmer by DLW and its magnetic drive; (**b**) the SEM image of micro-simulators; and (**c**) the response of the micro-simulators to external magnetic field [89].

The study demonstrated good responsiveness of the magneto-photonic structure to the magnetic field by fabricating a miniature array. Figure 19a shows the fabrication process of magnetic structures and a magnetic field driving process. The DLW method is used to prepare a series of micro-simulators in the middle of the substrate, which can move freely within the liquid. An external magnetic field is then applied by the permanent magnet to investigate the response of the magnetic micro-simulators. During the DLW process, only a few milliwatts of the 532 nm CW working laser is required to fabricate fine pattern of the miniature array, see Figure 19b. It is found that, after the application of the magnetic field, all the micro-flashers moved rapidly toward the magnetic tip as shown in Figure 19c. In addition, the researchers also prepared micro-fans and micro-springs using the DLW method. The micro-fan consists of a central strut and two blades similar to the fan-rotor, and the rotation of the fan can be controlled by applying external magnetic field. For the micro-spring, one of its end is fixed on the cubic anchor which is connected to the substrate, and the other end can freely move on the substrate with magnetic beads attached to the middle session. It is shown that, by applying LOPA DLW technology, many magneto-photonic microstructures or devices can be fabricated using magnetic polymer nanocomposites, which can be potentially applied in making magnetic nanodevices and micro-robots.

## 4. Polymer-Based Bio-Devices by DLW

The application of DLW technology is also widely used in bioengineering and biomedical applications for purposes as medical surgery, treatment, detection, diagnosis, drug screening, delivery, and analysis [39,90]. In this part, bio-related devices such as microfluidics, micro-network, microneedles, and bionic actuation structures are introduced.

### 4.1. Microfluidics by DLW

Polymer-based microfluidic channels has been widely used in bio-applications due to its good bio-compatibility, low cost and small volume for detection [91]. Shin et al. used femtosecond laser micro-scribing technology to design and fabricate DNA distributor on PDMS substrates with a linewidth less than 10 μm [92]. The micro-channels, which can be used to divide DNA strands with different length, provides an easy and cheap way to observe the size distribution of DNA in solution.

**Figure 20:**
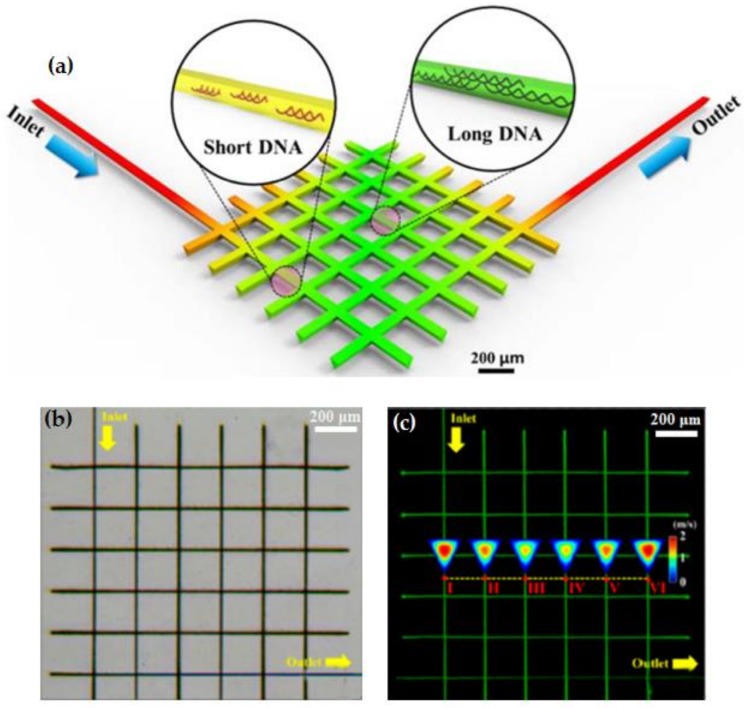
(**a**) Schematics of the DNA distributor; (**b**) tThe SEM image of the manufactured DNA distributor; and (**c**) fluorescence image of DNA solution distribution in each channel at a flow rate of 5 μL/min [92].

The DLW process for DNA distributor fabrication uses a femtosecond laser with a wavelength of 343 nm and a pulse time of 190 fs. Laser micro-scribing is performed on a 5 mm thick PDMS block. The inlet of the manufactured microfluidic system is connected to a syringe pump, and then the λDNA solution stained with YOYO-1 iodide dye is separately injected into the microfluidic channel at a flow rate of 1, 5 and 15 μL/min, as shown in Figure 20. The results show the correlation between the cross-sectional velocity distribution at the channels and the inlet flow rate of DNA solution. It can be found that, as the flow rate increases, the speed of the external channel increases faster than that of the intermediate channel. In addition, as the flow rate gets faster, this difference becomes more obvious. A fluorescence microscopy is used to distinguish the DNA distribution in this work, since brighter fluorescence intensity can be observed from longer DNA strands [93], as shown in Figure 20c. It can be verified that the relative light intensity of the intermediate channel is the strongest while the outer channel has weaker intensity at the same flow rate.

**Figure 21:**
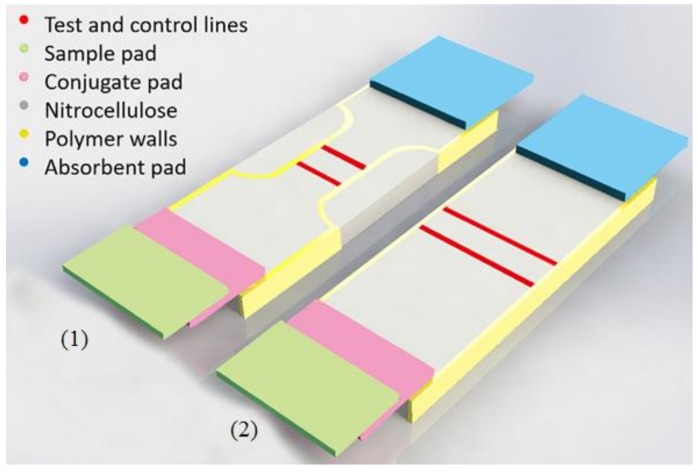
Schematic of a lateral flow device with and without polymer sidewalls [46].

The lateral flow device (LFD) is commonly used for detecting various biological analytes. Katis et al. proposed a polymer-based LFD prepared on a nitrocellulose membrane (a porous substrate) by DLW technology [46]. The photopolymer used is the acrylate-based Desolite 3471-3-14. The liquid photopolymer is dispensed in the reaction zone on the substrate and inscribed by a 405 nm CW diode laser. Schematic of the optimized LFD is shown in Figure 21, which is compared to a conventional LFD. The polymer solution is locally deposited on the reaction zone to form polymer microfluidic channels with different widths. With narrower width of the channel (Figure 21(1)), performance of the LFD can be improved with higher detection sensitivity and better detection limit of the analyte, quantified by C-Reactive Protein (CRP) sandwich assay.

The CRP detection limit of the LFD with 5 mm channel width is 150 ng/mL while for the 1 mm channel width, it is 5 ng/mL. Compared with the conventional LFD, the detection limit of DLW fabricated LFD decreases by 30 times. In addition, the detection sensitivity of the device can be improved by 62 times, which demonstrates the advantage of the DLW process applied in this work.

### 4.2. Micro-Network by DLW

**Figure 22:**
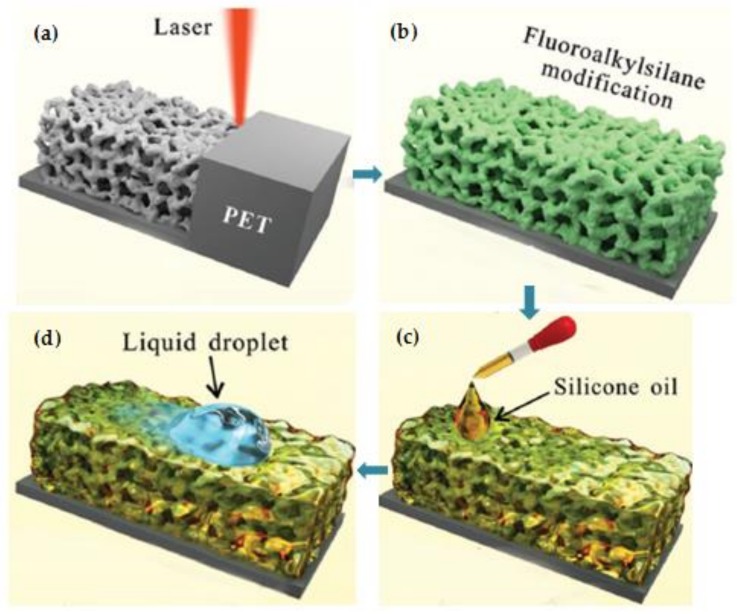
Schematic diagram of the preparation steps of slippery micro-network on PET [38].

Micro/nano-sized porous materials have attracted a lot of attention in the field of microstructure design and fabrication due to their large specific surface area, homogeneous and controllable pores, and diverse structures, which can be used in a wide range of fields, including optoelectronics, energy, environment, biomedicine and chemistry [94,95,96]. Yong’s group found that a one-step femtosecond laser ablation technique can be used to fabricate pores with the size of several hundred micrometers in diameter on a PET substrate, which can be adopted as a medium to promote cell reproduction [38]. If the porous surface is chemically treated, it is also possible to produce a slippery surface with excellent hydrophobic property which inhibits the growth of tumor cells. The fabrication steps of slippery micro-network surface are shown in Figure 22. The first step is laser ablation to form the micro-network, where a Ti: sapphire femtosecond laser with an emission wavelength of 800 nm is used to process 0.3 mm thick PET substrate. The ablated microporous structure is then immersed in a 0.5% fluoroalkylsilane solution for chemical treatment to reduce the surface free energy for better liquid repellence effect. After the chemical modification, a large amount of silicone oil is dropped on the inscribed porous structure to form a lubricating layer. It is found that a large variety of solution droplets will slide freely from the processed surface, showing an excellent liquid-repellent property.

The cell behavior at the cell-material interface can be affected by various factors, such as chemical composition of the material, surface morphology, wettability, charge distribution, and so on [97,98,99,100,101,102]. In this study, C6 glioma cells are inoculated on several different surfaces, including fluoroalkylsilane-modified PET surface, rough PET surface with laser ablation and the manufactured slippery surface to verify the improved performance of the fabricated micro-network. It can be found that the cell densities for the flat surface, laser processed rough surface and the fabricated slippery surface are 1349, 3469, and 21 mm^−2^, respectively, indicating a great improvement on the specific surface area and hydrophobic property. In addition, the process can be applied to a wider variety of other polymeric materials with the same parameters, such as PMMA, PA, PC, PE, and PLA, which can also achieve superior performance as porous network microstructures. In addition, DLW technology is also often used to process special polymer materials such as proteins and hydrogels to obtain functional micro-units that are non-toxic, harmless, and biocompatible. It can be applied to a large variety of biomedical related fields, including cell microenvironment culture, nerve fiber induction, and three-dimensional tissue culture [4,103,104,105].

### 4.3. Microneedles by DLW

For the past two decades, microneedles (MNs) have been investigated for the painless transmission of drugs through the stratum corneum [106]. Among many ways for the design and manufacturing of microneedles, DLW on polymer material has become a favorable method due to its simple processing procedure, large scale preparation and low cost. Organic polymer, which can be engineered with good biocompatibility properties, is considered as the ideal material for biomedical applications [107]. In addition, for the microneedle fabrication, it is also very important for the material to have higher strength. It has been reported that polymer material SU-8, a mixture of bisphenol A, triarylsulfonium hexafluoroantimonate, and cyclopentanone, has very good biocompatibility as well as strong strength [108,109], which makes it suitable for MN fabrication.

**Figure 23:**
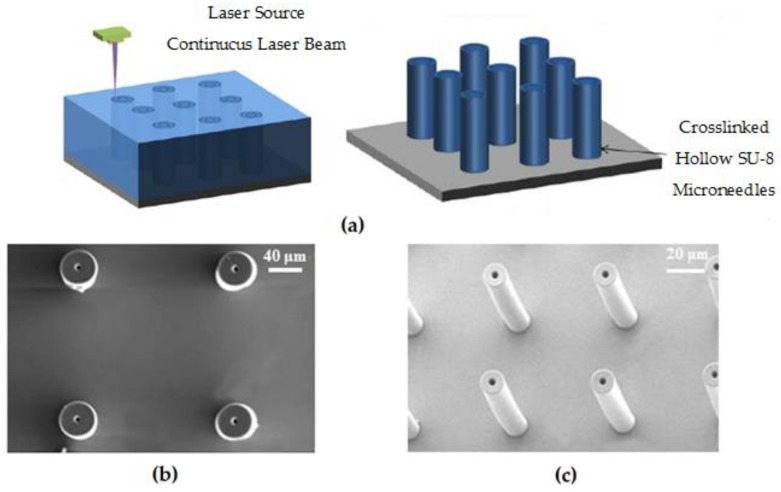
(**a**) Schematic diagram of DLW to fabricate SU-8 microneedle array; (**b**) the SEM top view of prepared SU-8 microneedle array; and (**c**) the SEM image of microneedle tilted at 20° [20].

Mishra et al. have designed and manufactured SU-8 cylindrical hollow-structure MNs for transdermal administration in this manner [20]. The schematic of the manufacturing process is shown in Figure 23a. A 500 μm thick SU-8 layer is deposited on a silicon substrate, and then inscribed using a helium-cadmium laser with a wavelength of 325 nm. After removing unexposed areas, a series of SU-8 microneedles with 550 μm in height, 110 μm, and 25 μm in outer and inner diameter can be obtained, as shown in Figure 23b,c. It can also be noticed that the manufactured microneedles are hollow cylindrical shapes without sharp tips, which greatly saves manufacturing time and can be suitable for mass production. To validate the effectiveness and repeatability of the prepared microneedles for the use of transdermal drug delivery, the device is mounted to a “T”-shaped aluminum plate, immersed in methylene blue, and then stamped onto the skin surface of an albino mouse. The results show that MNs can effectively pierce the mouse skin, and even if after 10 repeated piercing tests, the MNs do not break, suggesting very good strength and repeatability.

### 4.4. Bionic Actuation by DLW

**Figure 24:**
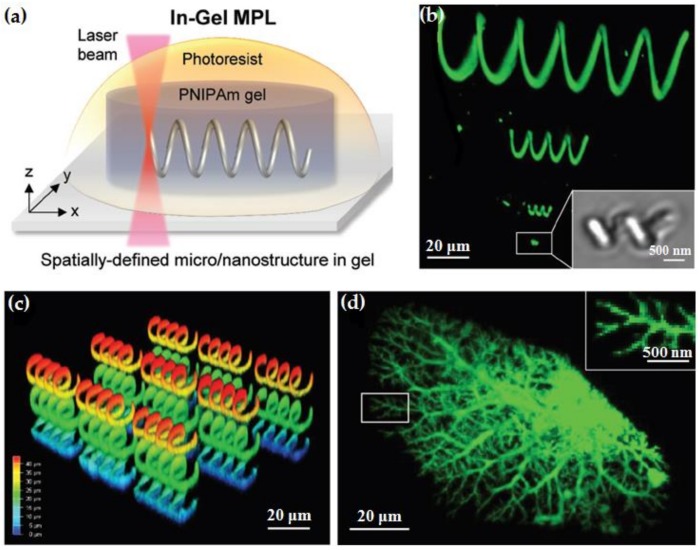
(**a**) Schematic diagram of spiral structure in hydrogel by DLW; The 3D-reconstructed CLSM image of (**b**,**c**) Spiral model structures; and (**d**) blood vessel model structure [110].

Self-forming and drivable materials inspired by the active motion in biological systems have broad application prospects in biosensors, soft robots, and bionic actuation. Nishiguchi’s group developed a method to prepare a freely suspended photosensitive resin microstructure using DLW multiphoton lithography technology in a hydrogel material [110], achieving a resolution as high as 1/100 nm. The response of the hydrogel to ambient humidity leads to a change in the restricted volume, resulting in bending and deformation movements, which can be used for soft actuators and artificial muscles [111,112,113,114,115]. The DLW technology can be suitable to make small micron-sized heterogeneous composite structures, which can achieve rapid hydraulic actuation by absorbing and releasing water in a short period of time. In addition, the direct written resin microstructure in the thermosensitive hydrogel produces a large motion effect with very small temperature variations. Figure 24a shows the preparation of spatially-defined micro/nanostructure in hydrogel using DLW method. A 50 μm thick heat-sensitive poly(N-isopropylacrylamide) (PNIPAm) gel film is prepared as the base material with a layer of photoresist, acrylate monomer (IP-L 780, n = 1.48), covering the top to make the gel expand by about 30%. The composite material is written by a femtosecond laser with an emission wavelength of 780 nm and an oil immersion objective of 63× (NA = 1.4), forming a spiral mciro/nano-structure. Figure 24b–d present different fabricated structures in PNIPAm hydrogel, verifying the feasibility of the DLW method. Furthermore, the bending and distortion of the controlled region which can be caused by the water absorption/releasing is also investigated. In order to testing the buckling deformation effect of the structure under thermal actuation, three slab structures are also written into the PNIPAm gel. It can be found that more complex motion patterns can be realized by developing new materials which can generate stress variation caused by environment changing. Apart from external temperature inducing actuation, Marc et al. found that local temperature changes caused by two-photon absorption effect of DLW also leads to a local actuation response of the PNIPAM-based 3D heterostructure [116].

## 5. Special Materials for DLW

In the fabrication of polymer-based 2D/3D microstructures using DLW technology, more materials that have interesting optical, opto-electronic, or ion-exchanging properties have been developed. This section will introduce three polymer materials which are used in DLW systems, including a low-cost photoinitiator for replacing expensive dye molecules, a luminescent material with excellent photoelectric property, and a negatively-charged ion-exchanger in aqueous solution.

**Figure 25:**
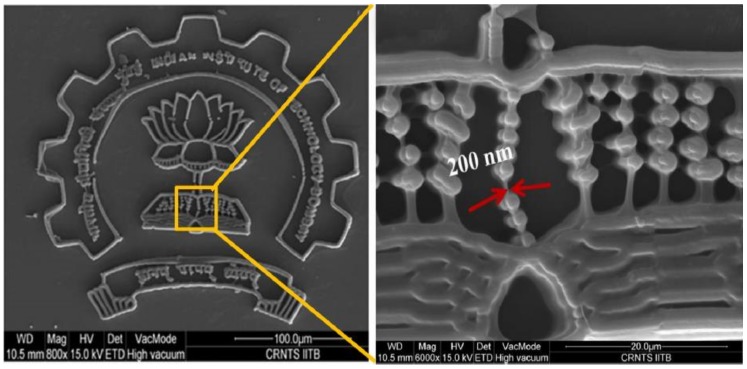
The SEM image of “Technology Bombay” logo with the line width of 200 nm by DLW [119].

In general, in order to achieve the two-photon absorption effect in the DLW process, it is necessary to add dye molecules which can absorb two (or more) photons while the sum of that energy will induce a transition in the molecule that, in turn, initiates the polymerization. However, the dye molecules are expensive, highly toxic, and less soluble in polymer materials, leading to lower efficiency and higher cost for the fabrication process [117]. In order to achieve sub-wavelength-scale direct writing without using dye molecules, a highly efficient photoinitiator can be applied to produce free radicals quickly and sensitively in small areas at low optical doses [118]. Chaudhary et al. have investigated the polymer material, Ethyl-2,4,6-Trimethyl-benzoylphenylphosphinate (Lucirin-TPO-L), as the high-dose two-photon photoinitiator additive to replace dye molecules, which is inexpensive and readily soluble in polymers [119]. Although its two-photon absorption coefficient is small, it has a high free radical quantum yield of 0.99 [120]. The study uses a mixture of two triacrylate monomer liquid resins, tris(2-hydroxyethyl)isocyanurate triacrylate (SR368) and ethoxylated (6) trimethylolpropane triacrylate (SR499) as the substrate material. The materials are mixed at a ratio of 50:50 by weight, and about 20 wt % of Lucirin-TPO-L is added into the mixture to enhance the high resolution (1/200 nm) of DLW process, as shown in Figure 25. It illustrates that the addition of a large amount of TPO-L photoinitiator enables sub-wavelength resolution structure writing without the use of dyes.

**Figure 26:**
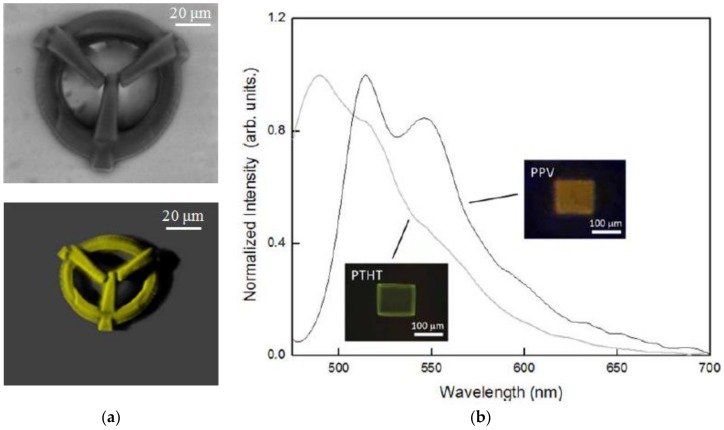
(**a**) The SEM and fluorescence images of PPV-doped microstructure; and (**b**) photoluminescence spectra of the same direct writing structure before (PTHT doped) and after thermal conversion (PPV doped) [123].

Another polymer material, poly(p-phenylene vinylene) (PPV), has shown broad application prospects in organic electroluminescence and photoluminescence due to its good processing properties as a polymer and its photoelectric properties as an organic semiconductor material [121,122]. It can be synthesized by thermal conversion from its precursor polymer poly(xylenetetrahydrothiophene chloride) (PTHT) which is easy to dissolve in organic solvent. Avila et al. combined the thermal conversion process with the fs laser two-photon polymerization (TPP) DLW technique to realize 3D PVV microstructures [123]. In this work, SR368 and SR499 are mixed at a ratio of 10:90 by weight together with 0.5 wt % of the precursor polymer PTHT and 3 wt % photoresist Lucirin-TPO-L. After the microstructure is fabricated, it is placed in a vacuum oven at 230 °C for heat conversion treatment for 6 h. Finally, the microstructures before and after thermal conversion are observed using SEM and z-stack confocal fluorescence microscopy, as shown in Figure 26a. It can be found that microstructures can be fabricated with fine features and low shrinkage factors. From the confocal fluorescence microscopy observation, it can be seen that the spatial distribution of PPV is very uniform, and the DLW process does not affect the optical properties of PPV material. The photoluminescence properties of the microstructures fabricated before and after thermal conversion is also characterized, see Figure 26b. After thermal conversion, the peak at 490 nm and the shoulder peak at 515 nm are red-shifted to 515 and 550 nm, which match the PPV characterization peaks, indicating the formation of PVV after the thermal conversion process.

**Figure 27:**
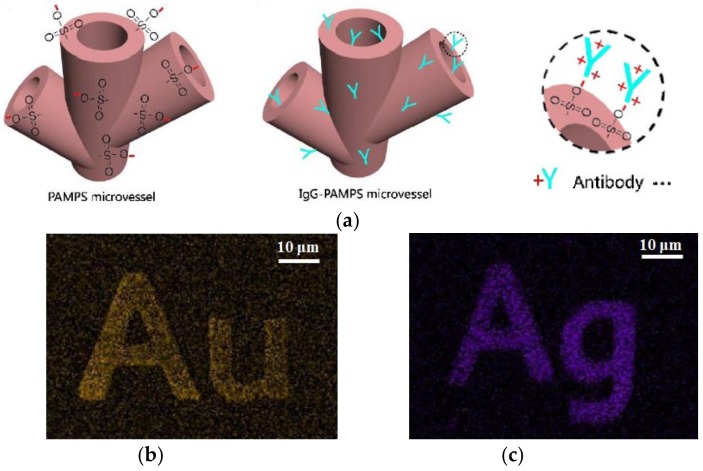
(**a**) Schematic diagram of electrostatic adsorption of 3D PAMPS microvessels with positively charged and positively charged IgG antibodies; (**b**) positively-charged Au nanorods adsorbed on a negatively charged “Au” PAMPS pattern; and (**c**) negatively-charged Ag nanosheets adsorbed on a positively charged “Ag” PAMPS pattern [124].

Wang et al. have proposed a method to functionalize microstructures by using ion-exchangeable polymer material [124]. Poly(2-acrylamido-2-methyl-1-propanesulfonic acid) (PAMPS) is adopted in this work to prepare ion-exchangeable microstructures by one-step DLW. PAMPS is an ion exchanger that can be ionized in a solution and directly written by a femtosecond laser to obtain a 3D microstructure. The DLW material is prepared using 2-acrylamido-2-methyl-1-propanesulfonic acid (AMPS monomer) as the prepolymer, poly(ethylene glycol) diacrylate (PEGDA) as the crosslinking agent and rhodamine B (RhB) as the photosensitizer. After the DLW process, the prepared microstructure is rinsed in water to obtain a charged ion-exchangeable microstructure. The scanned areas have sulfonic acid residue with rich negative charge which can be used for electrostatic adsorption of positively-charged matter, such as metal ions, nanoparticles, and proteins to form functionalized components. Figure 27a shows a 3D PAMPS microvascular structure prepared in this manner. The entire microcontainer is negatively charged which can adsorb proteins. The preparation of PAMPS microvessels not only realizes the simulation of biological morphology, but also realizes the simulation of biological function.

Figure 27b,c demonstrates the ability of the PAMPS microstructure to adsorb ionized nanoparticles. The Au nanorods are first modified with a positively-charged surfactant, cetyltrimethylammonium bromide (CTAB), and then adsorbed on the “Au” PAMPS pattern, as shown in Figure 27b. In addition, by immersing the “Ag” PAMPS pattern in a high concentration of ethylenediamine solution (positively-charged), the same device can be used to adsorb negatively-charged nanoparticles, for instance, modified Ag nanosheets with a negative charge, see Figure 27c. By using the same method, other kinds of charged materials can be adsorbed onto the PAMPS microstructure, which may have potential application for sensing purposes.

## 6. Conclusions and Outlook

Recent progress of using DLW technology for the preparation of polymer-based devices as well as their applications are reviewed in this work. By selecting suitable components, processing parameters and polymer materials for DLW systems, a series of microstructures and micro-devices can be obtained and used in various fields including photonic integration, microelectronics, electro-optical, magneto-photonic and bioengineering. A large variety of passive optical devices, such as polymer based fiber gratings, microresonators, microlenses, and optical waveguide couplers can be fabricated using the DLW technique with high resolution. In addition, electro-optic/magneto-photonic devices, such as all-photon electronically-controlled switching polarizers, electro-optic modulators, and magneto-photonic microstructures, can also be prepared with the same method. Due to the thermal stability and flexibility of polymer materials, circuits and microcapacitors can be fabricated on flexible substrates. Furthermore, the excellent biocompatibility of polymer material means it can be used to provide a platform for bioreactivity, identification, and treatment, including a series of micro-devices, such as microfluidic channel, micro-network, and micro-needles. We have also reviewed some polymer materials which have unique properties and can be used for DLW device fabrication.

DLW technology has been extensively used in the processing of polymer materials for various applications. However, in order to achieve integration of devices, create superior performance and realize various functions, many problems need to be solved. For example, in the field of optics, the device integration is still being extensively investigated. In the field of electromagnetics, more DLW-related physical/chemical mechanisms need to be developed and ingeniously utilized to achieve more direct and efficient fabrication of micro-devices with certain electrical/magnetic properties. In addition, in the bio-field, the processing efficiency and scanning method of the device also need to seek greater breakthroughs, which require a large amount of research work. Surprisingly, with continuous development of new polymer composite materials, DLW technology can not only be applied to polymer materials, but can also be widely used in organic molecules, dielectrics, metals, semiconductors, proteins, hydrogels, and other materials [125,126,127,128,129]. Furthermore, for the preparation of polymer-based microstructures, other techniques, such as photo-lithography with exposing masks, imprinting technique, and ink-jet printing technique, also have their own advantages [130,131,132,133,134]. It can be expected that innovation of the processing techniques and combination of the DLW method with other fabrication techniques can be used to process a wide range of new materials to make devices with high complexity, more functionality, and better performance, which will be applied in even wider fields. DLW technology is expected to become the processing technology leading the next generation of integrated devices.
